# Psychiatric Characteristics Across Individuals With *PTEN* Mutations

**DOI:** 10.3389/fpsyt.2021.672070

**Published:** 2021-08-17

**Authors:** Morgan Steele, Mirko Uljarević, Gaëlle Rached, Thomas W. Frazier, Jennifer M. Phillips, Robin A. Libove, Robyn M. Busch, Patricia Klaas, Julian A. Martinez-Agosto, Siddharth Srivastava, Charis Eng, Mustafa Sahin, Antonio Y. Hardan

**Affiliations:** ^1^Department of Psychiatry and Behavioral Sciences, Stanford University, Stanford, CA, United States; ^2^The School of Psychological Sciences, University of Melbourne, Melbourne, VIC, Australia; ^3^La Trobe University, Melbourne, VIC, Australia; ^4^Saint Joseph University, Faculty of Medicine, Beirut, Lebanon; ^5^Autism Speaks, New York, NY, United States; ^6^Department of Psychology, John Carroll University, University Heights, OH, United States; ^7^Genomic Medicine Institute, Cleveland, OH, United States; ^8^Neurological Institute, Cleveland Clinic, Cleveland, OH, United States; ^9^Departments of Human Genetics, Pediatrics and Psychiatry, University of California, Los Angeles, Los Angeles, CA, United States; ^10^Translational Neuroscience Center, Department of Neurology, Boston Children's Hospital, Harvard Medical School, Boston, MA, United States; ^11^Department of Genetics and Genome Sciences, Case Western University School of Medicine, Cleveland, OH, United States; ^12^Department of Neurology, Rosamund Stone Zander Translational Neuroscience Center, Boston Children's Hospital, Harvard Medical School, Boston, MA, United States

**Keywords:** *PTEN*, macrocephaly, autism, behavioral problems, internalizing and externalizing

## Abstract

Germline heterozygous *PTEN* mutations have been associated with high prevalence of autism spectrum disorder (ASD) and elevated rates and severity of broadly defined behavioral problems. However, limited progress has been made toward understanding whether *PTEN* mutation is associated with specific psychiatric co-morbidity profiles when compared to idiopathic ASD. The current study aimed to utilize a cross-measure approach to compare concurrent psychiatric characteristics across children and adolescents with *PTEN* mutation with (*PTEN*-ASD; *n* = 38) and without ASD (*PTEN*-No ASD; *n* = 23), and ASD with macrocephaly but no *PTEN* mutation (macro-ASD; *n* = 25) using the Child Behavior Checklist (CBCL) and the Aberrant Behavior Checklist (ABC). There were significant group effects for the CBCL Internalizing and Externalizing broad symptom score, the majority of specific CBCL syndrome scores, and all ABC subscale scores. *Post-hoc* comparisons revealed greater behavioral symptoms in the ASD groups (*PTEN*-ASD and macro-ASD) compared to the *PTEN*-no ASD group on nearly all subtest scores examined. There were no statistically significant differences between the *PTEN*-ASD and macro-ASD groups; however, there was a trend for the macro-ASD group showing higher levels of aggressive behaviors. Our findings provide evidence of specific behavior profiles across *PTEN*-No ASD, *PTEN*-ASD, and macro-ASD groups and highlight the importance of early identification of behavioral vulnerabilities in individuals with *PTEN* mutations in order to provide access to appropriate evidence-based interventions.

## Introduction

Germline mutations in the gene encoding phosphatase and tensin homolog tumor suppressor (*PTEN)* result in a range of physical, behavioral and cognitive features including macrocephaly, executive functioning deficits, elevated rates of intellectual disability and high prevalence of autism spectrum disorder (ASD) (1–4). Indeed, pathogenic *PTEN* mutations are identified in ~2% of all ASD cases and up to 20% of cases with ASD and macrocephaly (2, 5–7) with 17% weighted average reported across more than 10 studies (8). Furthermore, regardless of age, at least 23% of individuals with *PTEN* mutations meet DSM-5 diagnostic criteria for ASD (3, 9, 10), and research has started to explore whether individuals with *PTEN* mutations differ in terms of presentation and severity of core ASD symptoms from individuals with idiopathic ASD (2, 11). In addition, recent studies have suggested a high rate of behavioral difficulties in individuals with *PTEN* mutations across the lifespan (12). However, it remains unclear whether the profile of behavioral difficulties and neuropsychiatric symptoms varies between those with and without a comorbid ASD diagnosis and how it compares to that of individuals with idiopathic ASD.

Prior research has reported a variety of neuropsychiatric phenotypes in individuals with *PTEN* mutations (3, 13, 14). For instance, clinical presentations in a study of nine patients with PHTS ranged from asymptomatic macrocephaly to clinical diagnosis of anxiety, bipolar disorder, obsessive-compulsive disorder (OCD), psychosis and adult-onset movement disorder (13). Another retrospective chart review of 47 patients aged between 1 and 26 years of age showed that apart from ASD diagnosis, which was found in 50% of individuals, 34% of the cohort had at least one behavioral/psychological diagnosis with ADHD (24%) and anxiety (15%) being the most common (3).

In a recent study by our group, behavioral problems were examined across three patient groups: individuals with *PTEN* mutations with and without ASD diagnosis (*PTEN-ASD* and *PTEN*-no ASD) and individuals with idiopathic ASD with macrocephaly (macro-ASD) (2). No differences were found between the two ASD groups on the severity of broad internalizing and externalizing symptom spectra; however, the *PTEN*-no ASD group had significantly lower scores than both ASD groups. However, no comparison of the severity of more specific symptom domains within internalizing and externalizing spectra was conducted and the potential impact of cognitive level on detected group differences was not explored.

In summary, although evidence of a relationship between germline heterozygous *PTEN* mutations and ASD is well-established and research has shown elevated rates and severity of broadly defined behavioral problems, a significant knowledge gap exists in terms of the impact on the expression and fine-grained profile of psychiatric and behavioral symptoms and disruptive behaviors. Therefore, in the current study, we utilized a cross-measurement approach to increase our understanding of whether *PTEN* mutation with or without ASD is associated with specific comorbidity profiles when compared to idiopathic ASD, and whether individual variations in IQ affect these profiles.

## Methods

### Participants

Participants were recruited at four sites: Cleveland Clinic, Stanford University, University of California at Los Angeles, and Boston Children's Hospital. Individuals 3–21 years old with a clinical diagnosis of ASD with history of macrocephaly and individuals with or without a verified PTEN mutation without deleterious copy number variation beyond the *PTEN* region were invited to participate. ASD diagnoses were made based on the Autism Diagnostic Interview–Revised [ADI-R; (15)], Autism Diagnostic Observation Schedule, Second Edition [ADOS-2; (16)] and DSM-5 criteria. Individuals with ASD and macrocephaly, defined by a head circumference at or above the 98th percentile were included in the macro-ASD group. *PTEN*-No ASD participants were required to have scores below the broad spectrum cut-off on the ADOS-2, T scores below 60 on the Social Responsiveness Scale [SRS-2; (17)], and deemed not to have ASD by clinical evaluation. Participants with ASD and intellectual disability (IQ <70 with adaptive function deficits) were included in the study, but those with intellectual disability without ASD were excluded to reduce sample heterogeneity. Individuals with a clinically significant medical disease that would prohibit participation in the study procedures were excluded from the study. See Table 1 for the descriptive statistics of the sample.

**Table 1:** Demographic characteristics.

	***PTEN*-ASD**	**macro-ASD**	***PTEN*-no ASD**	**Statistics**
	***N* = 38**	***N* = 25**	***N* = 23**	
Age (Mean [SD])	8.93 (4.75)	11.99 (5.15)	8.94 (4.85)	*F* = 2.94, *p* = 0.057, *$\eta_{p}^{2}$* = 0.16
Sex (Number [%])				*χ2* = 3.56, *p* = 0.059, *v* = 0.20
Female	8 (21.1)	4 (16)	8 (34.8)	–
Male	30 (78.9)	21 (84)	15 (65.2)	–
FSIQ	65.90 (22.83)	71.27 (24.61)	96.92 (21.63)	*F* = 14.11, *p* <0.001, *$\eta_{p}^{2}$* = 0.29
Race (Number [%])		–		*χ2* = 1.82, *p* = 0.18, *v* = 0.29
White/Caucasian	30 (78.9)	14 (56)	12 (52.2)	–
Black/African American	1 (2.6)		–	–
Asian	–	5 (20)	3 (13)	–
Multiracial	5 (13.2)	5 (2)	6 (26.1)	–
Pacific Islander	–	1 (4)	–	–
Unknown/Not Reported	2 (5.3)	–	2 (8.7)	–
Ethnicity (Number [%])				*χ2* = 3.04, *p* = 0.55, *v* = 0.12
Hispanic	6 (15.8)	2 (8)	1 (4.3)	–
Not Hispanic	31 (81.6)	23 (92)	21 (91.4)	–
Unknown/Not Reported	1 (2.6)		1 (4.3)	–

Eighty-six participants met all inclusion/exclusion criteria and were included in this study. Participants were divided into the following three subgroups based on clinical diagnosis and the results of PTEN genotyping: ASD + *PTEN* mutation (*PTEN*-ASD; *n* = 38; Mage = 8.93 years, SD_age_ = 4.75), ASD with macrocephaly, no *PTEN* mutation (macro-ASD; *n* = 25; M_age_ = 11.99 years; SD_age_ = 5.15), and *PTEN* mutation without ASD (*PTEN*-no ASD; *n* = 23; M_age_ = 8.94 years; SD_age_ = 4.85).

### Measures

Participants' cognitive level was assessed using the Stanford Binet, Fifth Edition or the Mullen Scales of Early Learning (18, 19). Participants with a history of an ASD diagnosis received confirmatory diagnostic assessment with the Autism Diagnostic Interview- Revised (ADI-R) (15) and/or the Autism Diagnostic Observation Schedule- Second Edition (ADOS-2) (16). The ADI-R and ADOS-2 were administered by trained research staff and supervised by a research-reliable clinician.

The Child Behavior Checklist [CBCL; (20)] is a norm-referenced, parent-report questionnaire measure designed to assess behavioral, emotional, and social problems in children aged 1.5–5 years and 6–18 years (20). Each item is rated on a three-point Likert scale ranging from 0 (Not True) to 2 (Very True or Often True). In addition to total and Internalizing and Externalizing scores, both versions of the CBCL provide the following overlapping syndrome scores: Anxious/Depressed, Withdrawn, Somatic Problems, which constitute Internalizing Problems scores, and Attention Problems and Aggressive Problems, which constitute Externalizing Problems scores. In the present report, the *T* scores and combined specific overlapping subscales across the two different versions of the CBCL were used.

The Aberrant Behavior Checklist [ABC; (21)] is a 58-item rating scale used to assess a range of maladaptive behaviors including Irritability, Hyperactivity, Lethargy/Withdrawal, Stereotypy and Inappropriate Speech. Each item is rated on a four-point Likert scale ranging from 0 (not at all problem) to 3 (the problem is severe in degree). In this study, we did not utilize the Stereotypy scale given that the focus was on the profile of behavioral problems/co-occurring symptoms rather than on core ASD symptoms.

## Results

### Preliminary Analyses

There were no age, sex, race nor ethnicity differences between the three groups (Table 1). However, the *PTEN*-ASD and macro-ASD groups had significantly lower FSIQ (*F* = 14.11, *p* <0.001, ηp2 = 0.29) scores than the *PTEN*-no ASD group.

### Main Analyses

*CBCL:*Table 2 shows the percentage of individuals across the three clinical groups scoring in the normative, at-risk and clinical range across the CBCL syndrome scales. Twenty-eight and 24 percent of macro-ASD participants met the cut-off criteria for clinically significant Internalizing and Externalizing problems, respectively. In the *PTEN*-ASD group, 47.8% of participants scored in the clinical and 15.8% in at-risk range for Internalizing problems and 34.2% in the clinical range for Externalizing symptoms. In the *PTEN*-no ASD group, 30.4 and 21.7% of participants scored in the clinical range for Internalizing and Externalizing symptoms, respectively.

**Table 2:** Frequency of psychopathologies across *PTEN*-ASD, Macro ASD and *PTEN*-no ASD groups based on the CBCL cut-off scores.

	***PTEN*** **-ASD (%)**			**Macro ASD (%)**			***PTEN*** **-no ASD (%)**		
	**Normative**	**Risk**	**Clinical**	**Normative**	**Risk**	**Clinical**	**Normative**	**Risk**	**Clinical**
Anxious/depressed	80.6	16.7	2.7	75.0	20.8	4.2	82.6	13.0	4.4
Withdrawn	45.9	21.6	32.4	58.3	29.2	12.5	82.6	8.7	8.7
Somatic problems	63.2	15.8	21.1	84.0	0.0	16.0	78.3	4.3	17.4
Attention problems	44.7	26.3	28.9	58.3	8.3	33.4	78.3	8.7	13.0
Aggressive problems	94.7	5.3	0.0	68.0	12.0	20.0	91.3	0.0	8.7

*Anxious/Depressed, Withdrawn and Somatic Problems syndrome scales constitute Internalizing Problems Score; Attention Problems and Aggressive Problems syndrome scales constitute Externalizing Problems Score*.

There was a significant group effect for both Internalizing (*F* = 4.57, *p* = 0.013, ηp2 = 0.10) and Externalizing (*F* = 8.80, *p* <0.001, ηp2 = 0.18) problems scores with *post-hoc* comparisons showing that the *PTEN*-no ASD group had significantly lower Internalizing problems when compared to the *PTEN*-ASD group (*p* = 0.011, *d* = 0.06) and significantly lower Externalizing problems than both the *PTEN*-ASD (*p* = 0.003, *d* = 0.07) and macro-ASD groups (*p* <0.001, *d* = 0.08). When examining group effects on the syndrome scales, there were significant effects for Withdrawn (*F* = 6.46, *p* = 0.003, ηp2 = 0.14), Attention Problems (*F* = 4.09, *p* = 0.020, ηp2 = 0.09), and Aggressive Problems (*F* = 4.27, *p* = 0.017, ηp2 = 0.09), but not for Anxious/Depressive or Somatic Complaints. *Post-hoc* analysis showed that while both macro-ASD and *PTEN*-ASD groups had higher Withdrawn scores than *PTEN*-no ASD, differences reached significance only for the macro-ASD vs. *PTEN*-no ASD contrasts for Attention and Aggressive Problems syndrome scales. See Table 3 for descriptives.

**Table 3:** Comparison of CBCL and ABC score severity across *PTEN*-ASD, Macro ASD and *PTEN*-no ASD groups.

	***PTEN*-ASD^a^ Mean (SD)**	**macro-ASD^b^ Mean (SD)**	***PTEN*-no ASD^c^ Mean (SD)**	**Statistics**	***Post hoc***			
					**Significant Contrasts**	**Cohen's d**		
						**a vs. b**	**a vs. c**	**b vs. c**
**CBCL**								
Internalizing	61.21 (9.08)	59.42 (8.56)	52.65 (15.07)	*F* = 4.57, *p* = 0.013, *$\eta_{p}^{2}$* = 0.10	a > c	0.20	0.69	0.55
Externalizing	53.13 (7.75)	55.75 (11.19)	43.56 (13.68)	*F* = 8.80, *p* <0.001, *$\eta_{p}^{2}$* = 0.18	a, b > c	0.27	0.86	0.97
Anxious/depressive	56.92 (6.41)	56.71 (7.44)	54.83 (7.29)	*F* = 0.70, *p* = 0.50, *$\eta_{p}^{2}$* = 0.02	NA	0.03	0.30	0.25
Withdrawn	65.16 (9.59)	63.83 (6.23)	57.17 (9.03)	*F* = 6.46, *p* = 0.003, *$\eta_{p}^{2}$* = 0.14	a, b > c	0.16	0.86	0.86
Somatic complaints	61.16 (8.61)	57.64 (10.24)	58.87 (10.14)	*F* = 1.10, *p* = 0.34, *$\eta_{p}^{2}$* = 0.03	NA	0.37	0.24	0.13
Attention problems	65.03 (10.11)	66.46 (11.24)	58.65 (8.77)	*F* = 4.09, *p* = 0.020, *$\eta_{p}^{2}$* = 0.09	b > c	0.13	0.67	0.77
Aggressive problems	54.71 (5.30)	59.20 (9.05)	53.43 (8.22)	*F* = 4.27, *p* = 0.017, *$\eta_{p}^{2}$* = 0.09	b > c	0.60	0.18	0.67
**ABC**								
Irritability	8.08 (7.42)	9.63 (7.49)	4.30 (7.60)	*F* = 3.20, *p* = 0.042, *$\eta_{p}^{2}$* = 0.07	b > c	0.21	0.50	0.71
Lethargy	11.19 (9.44)	10.85 (5.05)	3.91 (5.91)	*F* = 7.80, *p* <0.001, *$\eta_{p}^{2}$* = 0.16	a, b > c	0.08	0.92	1.26
Hyperactivity	12.16 (8.90)	13.67 (7.85)	7.22 (9.16)	*F* = 3.74, *p* = 0.028, *$\eta_{p}^{2}$* = 0.08	b > c	0.18	0.55	0.76
Inappropriate speech	3.40 (2.66)	3.33 (2.74)	1.70 (2.28)	*F* = 3.55, *p* = 0.033, *$\eta_{p}^{2}$* = 0.08	a > c	0.02	0.69	0.65

*ABC, Aberrant Behavior Checklist; CBCL, Child Behavior Checklist; CBCL Anxious/Depressed, Withdrawn and Somatic Problems syndrome scales constitute Internalizing Problems Score; CBCL Attention Problems and Aggressive Problems syndrome scales constitute Externalizing Problems Score*.

a *denotes PTEN-ASD group*,

b *denotes macro-ASD group and*

c *denotes PTEN-no ASD group*.

*ABC:* There were significant effects for all ABC subscale scores (Irritability [*F* = 3.20, *p* = 0.042, ηp2 = 0.07], Lethargy [*F* = 7.80, *p* <0.001, ηp2 = 0.16], Hyperactivity [*F* = 3.74, *p* = 0.028, ηp2 = 0.08] and Inappropriate Speech [*F* = 3.55, *p* = 0.033, ηp2 = 0.08]) with *post-hoc* analysis showing that the *PTEN*-no ASD group had significantly lower scores than (i) both ASD groups on Lethargy, (ii) macro-ASD on Irritability and Hyperactivity, and (iii) *PTEN*-ASD on Inappropriate Speech.

*Adjustment for FSIQ*: Given that the *PTEN*-ASD and macro-ASD groups had significantly lower FSIQ (*F* = 14.11, *p* <0.001, ηp2 = 0.29) scores than the *PTEN*-no ASD group, a series of univariate models were conducted to explore the contribution of FSIQ to the noted differences in CBCL and ABC scores. After adjusting for FSIQ score, there were still significant, albeit decreased in terms of effect size group differences for CBCL Internalizing (*F* = 3.28, *p* = 0.043, ηp2 = 0.08; Covariate; FSIQ: *p* = 0.68, ηp2 = 0.002) and Externalizing (*F* = 5.05, *p* = 0.009, ηp2 = 0.13; Covariate; FSIQ: *p* = 0.076, ηp2 = 0.05) problems, CBCL Aggressive Problems (*F* = 4.94, *p* = 0.01, ηp2 = 0.12; Covariate; FSIQ: *p* = 0.20, ηp2 = 0.02), and ABC Lethargy (*F* = 5.19, *p* = 0.008, ηp2 = 0.13; Covariate; FSIQ: *p* = 0.11, ηp2 = 0.03). Group differences were no longer significant for CBCL Withdrawn (*F* = 1.96, *p* = 0.15, ηp2 = 0.05; Covariate; FSIQ: *p* = 0.20, ηp2 = 0.02), CBCL Attention Problems (*F* = 1.15, *p* = 0.32, ηp2 = 0.03; Covariate; FSIQ: *p* = 0.37, ηp2 = 0.01), ABC Irritability (*F* = 1.94, *p* = 0.15, ηp2 = 0.05; Covariate; FSIQ: *p* = 0.007, ηp2 = 0.10), ABC Hyperactivity (*F* = 1.69, *p* = 0.19, ηp2 = 0.05; Covariate; FSIQ: *p* = 0.01, ηp2 = 0.09), or ABC Inappropriate Speech (*F* = 1.55, *p* = 0.22, ηp2 = 0.04; Covariate; FSIQ: *p* = 0.17, ηp2 = 0.03).

## Discussion

The current study aimed to describe specific problem behavior profiles in individuals with germline pathogenic mutations in the *PTEN* gene. Individuals with *PTEN*-ASD were compared with individuals with *PTEN*-no ASD and those with macro-ASD on broad Internalizing and Externalizing spectra, as well more specific symptom clusters. In addition, we aimed to investigate the contribution of cognitive functioning on the problem behavior profiles across these three clinical groups.

We first explored differences between individuals with ASD but with different genetic status. No significant differences were found between *PTEN*-ASD and macro-ASD groups in Internalizing or Externalizing Problems, nor on any of the syndrome subscales of the CBCL nor the ABC subscales. These findings are consistent with previous investigations (2) and extend prior findings to a greater level of specificity. While behavior comparisons of *PTEN*-ASD and macro-ASD subgroups are quite limited within the literature, prior analyses have suggested that the *PTEN*-ASD subgroup displays less emotion dysregulation than other ASD groups (22). Furthermore, clinical observations have described these individuals as being generally happy and more flexible with changes in environment and routine than those with idiopathic ASD (2, 11), in line with our finding that there is approximately a half-standard deviation difference between the groups. Our findings that for internalizing Problems, *PTEN*-ASD group (but not MACRO-ASD group) had significantly higher scores than *PTEN*-no ASD group, and that for Attention Problems and Aggressive Problems, Macro-ASD group (but not *PTEN*-ASD group) had significantly higher scores than *PTEN*-no ASD group, provide some support to the noted subjective clinical observations. However, this study may not have been sufficiently powered to detect some of the key differences, and future longitudinal time points may be needed to identify smaller profile differences.

The influence of ASD diagnosis on problem behavior expression was also examined. Individuals in the *PTEN*-no ASD group had significantly lower scores than both ASD groups for CBCL Internalizing, Externalizing, Withdrawn, Attention, and Aggressive Problems scores and for ABC Irritability, Lethargy, Hyperactivity and Inappropriate Speech scores. Differences on CBCL domains such as Anxious/Depressed and Somatic Problems scales were not significant. Although the majority of scores were in the normative range across all CBCL scales, it is important to note that apart for the CBCL Anxious/Depressed scale (where only 4.4% were in the clinical range), at least 8.7% of individuals with *PTEN*-no ASD had clinically significant behavior problems, particularly in terms of Somatic (17.4%) and Attention Problem (13%) syndrome scales and both Internalizing (30.4%) and Externalizing (21.7%) problems, a significantly higher rate of noted problems in the general population (23). These observations indicate that some, perhaps all, individuals with *PTEN* mutations, regardless of their ASD status, will also require clinical assessment and, if positive, treatment. Finally, differences between groups remained significant, albeit with reduced effect sizes, after adjusting for FSIQ scores.

This investigation has several limitations. The sample size of the different groups is small, which is related to the low prevalence rates of *PTEN* mutations. Larger studies are warranted to confirm the current observations. Additionally, findings reported here are based on parent-report measures, and future investigations should use a multi-method assessment approach and include clinician-administered standardized assessments. Further, despite clinical utility and robust psychometric properties of the ABC and the CBCL in ASD, it is important to highlight the fact that the presence of ASD is associated with atypical expression of psychiatric and behavioral symptoms, in particular anxiety and depression (24–27). Therefore, it will be important for future studies to include instruments such as the Anxiety Disorders Interview Schedule—Autism Addendum (28), the Parent-Rated Anxiety Scale for Youth With Autism Spectrum Disorder (29) or the Anxiety Scale for Children with Autism Spectrum Disorder (30) to ensure that these potentially important and clinically impactful symptoms are not missed or under-sampled. It will also be important to investigate continuities and discontinuities in the presentation and structure of neurodevelopmental and neuropsychiatric symptoms in neurogenetic syndromes when compared to other clinical groups.

Despite noted limitations, reported findings have important clinical implications. More specifically, elevated levels of behavioral problems in individuals with *PTEN* mutations highlight the importance of early identification of behavioral vulnerabilities to facilitate access to appropriate evidence-based interventions. Importantly, our findings also highlight areas for future investigations, including the mechanisms underlying behavioral problems. In particular, it will be crucial to utilize a multi-level approach to fully characterize the associations between *PTEN* pathway proteins with individual differences in cognitive, behavioral, and clinical profiles (31). In addition, prospective research should attempt to further understand the impact of both cognitive functioning and ASD traits and explore the role of factors such as emotion regulation, cognitive control and intolerance of uncertainty that have been shown as key risk factors across a range of psychopathologies, in both general clinical (32–34), ASD (35–41) and most recently *PTEN* (42) cohorts. This critical step will be essential in order to inform the development and personalized delivery of effective treatments.

## Data Availability Statement

The raw data supporting the conclusions of this article will be made available by the authors, without undue reservation.

## Ethics Statement

The study received IRB approvals from relevant sites that participated: Cleveland Clinic, Stanford University, University of California at Los Angeles, and Boston Children's Hospital. Written informed consent to participate in this study was provided by the participants' legal guardian/next of kin.

## Author Contributions

CE, MSa, TF, and AH designed the study. CE, MSa, AH, RB, PK, SS, and JM-A collected the data. MU, GR, and MSt had full access to the data and conducted the analyses. MSt and MU drafted the initial manuscript. All authors critically reviewed and provided the feedback on the initial version of manuscript and approved the final version of the manuscript.

## Consortium Affiliations

- Simon K. Warfield, PhD, Department of Radiology, Boston Children's Hospital, Boston, MA, United States.
- Benoit Scherrer, PhD, Department of Radiology, Boston Children's Hospital, Boston, MA, United States.
- Kira Dies, ScM, CGC, Department of Neurology, Boston Children's Hospital, Boston, MA, United States.
- Rajna Filip-Dhima, MS, Department of Neurology, Boston Children's Hospital, Boston, MA, United States.
- Amanda Gulsrud, PhD, UCLA Semel Institute for Neuroscience & Human Behavior, David Geffen School of Medicine at UCLA, Los Angeles, CA, United States.
- Ellen Hanson, PhD, Department of Developmental Medicine, Boston Children's Hospital, Boston, MA, United States.

## Conflict of Interest

The authors declare that the research was conducted in the absence of any commercial or financial relationships that could be construed as a potential conflict of interest.

## Publisher's Note

All claims expressed in this article are solely those of the authors and do not necessarily represent those of their affiliated organizations, or those of the publisher, the editors and the reviewers. Any product that may be evaluated in this article, or claim that may be made by its manufacturer, is not guaranteed or endorsed by the publisher.
